# Raised water temperature enhances benthopelagic links via intensified bioturbation and benthos-mediated nutrient cycling

**DOI:** 10.7717/peerj.17047

**Published:** 2024-02-28

**Authors:** Eilish M. Farrell, Andreas Neumann, Jan Beermann, Alexa Wrede

**Affiliations:** 1 Benthic Ecology, Alfred Wegener Institute, Helmholtz Centre for Polar and Marine Research, Bremerhaven, Bremen, Germany; 2 Aquatic Nutrient Cycles, Helmholtz Centre Hereon, Geesthacht, Hamburg, Germany; 3 Shelf Sea System Ecology, Alfred Wegener Institute, Helmholtz Centre for Polar and Marine Research, Helgoland, Germany

**Keywords:** Sediment reworking, Mudflat, Wadden Sea, Mesocosm, Ecosystem function

## Abstract

Sediment reworking by benthic infauna, namely bioturbation, is of pivotal importance in expansive soft-sediment environments such as the Wadden Sea. Bioturbating fauna facilitate ecosystem functions such as bentho-pelagic coupling and sediment nutrient remineralization capacities. Yet, these benthic fauna are expected to be profoundly affected by current observed rising sea temperatures. In order to predict future changes in ecosystem functioning in soft-sediment environments like the Wadden Sea, knowledge on the underlying processes such as sediment reworking, is crucial. Here, we tested how temperature affects bioturbation and its associated ecosystem processes, such as benthic nutrient fluxes and sediment oxygen consumption, using luminophore tracers and sediment incubation cores. We used a controlled mesocosm experiment set-up with key Wadden Sea benthos species: the burrowing polychaetes *Arenicola marina* and *Hediste diversicolor*, the bivalve *Cerastoderma edule*, and the tube-building polychaete *Lanice conchilega*. The highest bioturbation rates were observed from *A. marina*, reaching up to 375 cm^2^yr^−1^; followed by *H. diversicolor,* with 124 cm^2^yr^−1^ being the peak bioturbation rate for the ragworm. Additionally, the sediment reworking activity of *A. marina* facilitated nearly double the amount of silicate efflux compared to any other species. *Arenicola marina* and *H. diversicolor* accordingly facilitated stronger nutrient effluxes under a warmer temperature than *L. conchilega* and *C. edule.* The oxygen uptake of *A. marina* and *H. diversicolor* within the sediment incubation cores was correspondingly enhanced with a higher temperature. Thus, increases in sea temperatures may initially be beneficial to ecosystem functioning in the Wadden Sea as faunal bioturbation is definitely expedited, leading to a tighter coupling between the sediment and overlying water column. The enhanced bioturbation activity, oxygen consumption, and facilitated nutrient effluxes from these invertebrates themselves, will aid in the ongoing high levels of primary productivity and organic matter production.

## Introduction

Bioturbation is a transport process which has gained traction in recent decades due to its profound effect on ecosystem functioning within soft-sediment habitats around the world (Gogina et al., 2020; Farrell et al., 2023; Kristensen et al., 2012; Lardies et al., 2001; Lohrer, Thrush & Gibbs, 2004; Meysman, Middelburg & Heip, 2006; Nicholaus et al., 2019; Reise et al., 2010; Wrede et al., 2017). As a prime example for the importance of bioturbation, the expansive mudflats of the Wadden Sea act as a large biological filter, with ecosystem functions such as primary productivity, bentho-pelagic coupling, and remineralization being facilitated by bioturbation (Reise et al., 2010). Animal behaviours causing bioturbation are sediment reworking, burrowing, feeding and defecating activities (Kristensen et al., 2012). Benthic fauna further facilitate the transport of water across the sediment-water interface, carrying out a transport process delineated from bioturbation: bioirrigation. Both bioturbation and bioirrigation can have a profound influence on ecosystem processes (Braeckman et al., 2010; Gogina et al., 2020; Wrede et al., 2019a; Wrede et al., 2017), for example, bioturbation can either destabilize or stabilize sediments (Graf & Rosenberg, 1997; Grant & Daborn, 1994), thus affecting sediment shear strength (Meadows, Tait & Hussain, 1990), erosion rates (Harris et al., 2015), sediment resuspension (Davis, 1993) and sediment movement (Reise, 2002). Moreover, bioturbation affects the movement of microphytobenthos, nutrients, and organic matter into the water column (Fernandes, Sobral & Costa, 2006; Reise, 2002; Underwood & Paterson, 1993). Bioturbators can either enhance the burial and preservation of organic carbon in deeper sediment layers, thereby increasing the quality and amount of food available (Braeckman et al., 2011; Zhang et al., 2019), or elevate mineralization rates to decrease organic carbon content in sediment.

The introduction of oxygenated water by means of bioirrigation strongly enhances the delivery of oxygen into anoxic sediment layers (Forster et al., 1999). Thus, bioirrigation facilitates aerobic reactions in deeper parts of the sediment, and enhances the surface area upon which aerobic reactions can occur (Lohrer, Thrush & Gibbs, 2004; Wrede et al., 2018). The bioirrigative flushing by benthos also creates strong concentration gradients between burrow walls and porewater (Wenzhöfer & Glud, 2004), transporting compounds such as silicate (SiO${}_{4}^{4-}$), ammonium (NH${}_{4}^{+}$), nitrate (NO${}_{3}^{-}$), and nitrite (NO${}_{2}^{-}$), into the overlying water column. Aerobic sediment metabolism that is simulated by the process of bioturbation itself can even be larger than the oxygen consumption from the bioturbating animals alone (Glud, 2008). Accordingly, bioturbators can be considered as vital contributors to the ecosystem functioning of shallow shelf sea systems like the Wadden Sea (Griffiths et al., 2017; Meysman, Middelburg & Heip, 2006).

Over the past 50 years, water temperatures in the western Wadden Sea have increased by 2°C (Beukema & Dekker, 2020). Temperatures in August range between 16.3 and 21.9 °C (Martens & Van Beusekom, 2008; Van Aken, 2008), and the mean August temperature for the Sylt-Rømø Bight is 18 °C (Amorim et al., 2023). Temperature change is known to have wide-ranging impacts on benthic fauna, including limiting growth (Wood et al., 2010), changing sediment nutrient uptake (Nedwell & Walker, 1995; Osinga et al., 1996; Wood et al., 2010), as well as feeding, bioirrigation, and bioturbation capacities (Berkenbusch & Rowden, 1999; Hollertz & Duchêne, 2001; Meadows & Ruagh, 1981; Mugnai et al., 2003; Ouellette et al., 2004; Roskosch et al., 2012; White, Klahr & Robbins, 1987). Thus, it is crucial to assess how the behaviour of benthic species may be affected by temperature change in order to understand future ecosystem functionality of coastal environments like the mudflats of the Wadden Sea.

Here, we chose four intertidal key species with differing bioturbation and bioirrigation behaviours to test the effect of temperature on bioturbation and associated ecosystem functions: the polychaetes *Hediste diversicolor* (Müller, 1776), *Arenicola marina* (Linnaeus, 1758) and *Lanice conchilega* (Pallas, 1766) as well as the bivalve *Cerastoderma edule* (Linnaeus, 1758). The biodiffusors *H. diversicolor* and *C. edule* conduct constant random particle movements over short distances (Kristensen et al., 2012), whereas the polychaetes *A. marina* and *L. conchilega* can be classed as conveyors as they transport sediment particles from depth, to the sediment surface (or vice versa) (Kristensen et al., 2012).

As a surficial biodiffusor, *C. edule* conducts strong bioturbation movements due to its ploughing movements and shaking behaviour (Flach, 1996), in the top few centimeters of the sediment. Meanwhile, *H. diversicolor* creates and irrigates mucus lined Y- shaped burrows up to 15 cm deep (Christensen, Vedel & Kristensen, 2000; Kristensen, 1983), thus converting surface particles into structured matrices (Hedman et al., 2011; Mermillod-Blondin et al., 2004).

*L. conchilega* is sedentary and lives in a tube transporting particles also from the surface downwards, whereas *A. marina* selectively deposit feeds below the sediment surface and transports sediment *via* faecal piles at the top end of its burrow (Riisgard & Banta, 1998). Notwithstanding their more sedentary nature, both *A. marina* and *L. conchilega* introduce large amounts of water through irrigation in the sediment. *Arenicola marina* actively draws water from the overlying water column down into its burrow which can extend up to 40 cm deep into the sediment (Riisgard & Banta, 1998), and *L. conchilega* irregularly carries out high levels of water exchange through the motion of emerging and retreating into its tube, acting like an engine’s piston pump (Forster & Graf, 1995).

To understand how these key species influence ecosystem processes such as benthic fluxes and bioturbation under different temperature regimes, we tested how a temperature change from 15 to 20 °C could affect these bioturbating organisms. For this purpose, we measured their sediment reworking rates, as well as consequential nutrient and oxygen exchanges simultaneously in a mesocosm tank experiment.

## Materials & Methods

### Sediment & fauna sampling

Sediment was collected from two sites off the coast of List, on the island of Sylt (North Sea) in August 2020. The first site was located in Königshafen beyond the small island of Uthörn (55°02′19.3″N 8°24′28.7″E), henceforth referred to as the ‘Uthörn site’. The second site was adjacent to a *Lanice* reef, also in Königshafen (55°01′34.1″N 8°26′07.6″E), henceforth referred to as the ‘Reef site’. Surficial intertidal sediment (< 5 cm depth) was dug up with a spade, and sieved through a 1000 µm sieve into Plexiglas cores (height = 32 cm, ø= 10 cm) in order to remove all macrofauna. These defaunated sediment cores were transferred back to the facilities of the Wadden Sea Station of the Alfred Wegener Institute, Helmholtz Centre for Polar and Marine Research (AWI), and left for 24 h to allow the fine fraction of the sediment to settle and be retained. Any overlying water was thereafter decanted, and the sediment height adjusted in all cores to 17 cm (±1 cm).

The animals were collected 1 day later. *Arenicola marina* and *C. edule* were collected from the Uthörn site, while *L. conchilega* were collected from a Lanice reef at the Reef site. Individuals of *H. diversicolor* were observed at the Uthörn site, yet due to tidal time restrictions, were collected at a site further down the coast from the Uthörn site (54°59′49.5″N 8°22′55.5″E), from within burrows in the top 15 cm of sediment. *Arenicola marina* was sampled by digging up sediment (to 15 cm deep) adjacent to faecal piles and irrigation holes. Intact worms were retrieved and placed into storage aquaria. *Cerastoderma edule* were collected by hand from surface sediments (< 5 cm). Specimens of *L. conchilega* were collected from a Lanice reef adjacent to the Reef site. *Lanice* tubes were exposed by levering a spade, carefully removed from the sediment, and visually checked to exclude empty tubes. Animals were transferred back to the field station, and aeration stones were added into all storage aquaria during transport.

All specimens shared similar biomass within each species group. *Arenicola marina* were all between 7 and 10 cm. All collected individuals of *C. edule* were approximately two cm long (shell width). The *L. conchilega* specimens could not be measured lengthwise without removing them from their tubes, so adult animals were chosen on their tube diameter (at least two mm, according to Carey, 1987). Due to availability of animals, individuals of *H. diversicolor* had a wider size range (0.07–0.17 g wet weight).

### Experimental set-up and incubation

The experiments were performed in the mesocosm facility of the Wadden Sea Station. Six open top sediment cores were submerged into each of the 12 mesocosm tanks (72 incubation cores in total), which had a continuous incoming seawater flow from the Sylt-Rømø Bight (see Pansch et al., 2016 for a detailed description of the mesocosm facility). The tanks were continuously bubbled with oxygen pipes, and each core was supplied with an aeration stone. Aeration was kept below a level that could cause any sediment resuspension. Six of the mesocosm tanks had temperatures set to 15 °C (±0.5 °C), while 6 of the tanks had their temperature set to 20 °C (±0.5 °C). 15 °C was chosen as a first temperature treatment as a comfortable representation of temperature prior to the summer peak (Amorim et al., 2023), while 20 °C was chosen as a second temperature treatment to reach closer to the higher end of the temperature range for the area. Additionally, some dominant benthic species of the German Bight, such as *A. marina*, are known to exhibit physiological temperature limits of 21 °C (Sommer, Klein & Pörtner, 1997), so in order to ensure normal behaviour the higher temperature treatment was limited to 20 °C.

Salinity, pH, and temperature of the incoming water from the Sylt-Rømø Bight was measured before adjustment to the temperature of the tanks. Over the course of the experiment, the average water temperature of the Sylt-Rømø Bight was 19.1 °C, and on the day of sampling the animals, 20.5 °C (Table S1).

Shortly after fauna sampling, species-specific individuals were randomly allocated to respective sediment cores within each mesocosm tank. Accordingly, each mesocosm tank yielded four species cores, namely an *Arenicola* core, *Cerastoderma* core, *Hediste* core, and a *Lanice* core; as well as two sediment control cores for the Uthörn and Reef sediment controls. A single *A. marina* worm was deposited into each *Arenicola* core, 6 *C. edule* individuals were placed into each *Cerastoderma* core, and 5 *L. conchilega* tubes containing worms were ‘planted’ into the sediment cores, using the technique described by Ziegelmeier (1969). The collected *H. diversicolor* showed a higher variation in body size, therefore 1 ‘large’ individual and 3 ‘small’ individuals were chosen for each *Hediste* core. All species densities reflected natural densities for the German and Dutch Wadden Sea (Beukema, 1974; Flach, 1996; Flach, 1992a; Flach, 1992b; Gilbert et al., 2021; Rabaut et al., 2007; Reise, Simon & Herre, 2001). Twelve hours after animals were placed in the sediment cores, all cores were checked to ensure that the animals had remained burrowed. This is especially important for *C. edule*, where individuals can often become infected with a parasite causing them to remain on the sediment surface (Thieltges, 2006).

From the point of addition to the mesocosms, the animals were left to acclimate for 36 h, before cores were lifted from the mesocosm tank and a homogenized suspension (15 ml) of luminophores was added in an even layer across the sediment surface (4 g pink colour, 60 µm, 4 g green colour, 80–250 µm; Partrac Ltd UK). Cores were left out of the mesocosm tank until the luminophores settled, no longer than 3 min, before being resubmerged, starting the experiment.

Animals were incubated in the mesocosm tanks for a total of 10 days. On day 8 and 9, oxygen (O_2_) and nutrient flux measurements were conducted (see below). These measurements had to be split across 2 days due to the sheer number of replicates. On day 10, the cores were removed from their mesocosm tanks in the same order that the luminophores were added, to take bioturbation measurements (see below). Subsequently, animals were recovered from the cores and wet mass, dry mass, and ash free dry mass (AFDM) per core were measured (Table S2). The entirety of each whole individual specimen was muffled (*i.e.,* the entire muscle and shell mass of *C. edule* for example), at 500 °C for 5 h. Lastly, sediment samples were also taken from the incubation cores to measure the grain size from the two sites, and frozen.

During the course of the experiment, a single lugworm had escaped from its incubation core, and buried in the *Lanice* core within the same tank. In addition, one worm from another *Hediste* core had died within the top layer of the sediment. Therefore, these three replicates were excluded from any analysis (Table S2).

### Nutrient flux measurements

**Figure 1:**
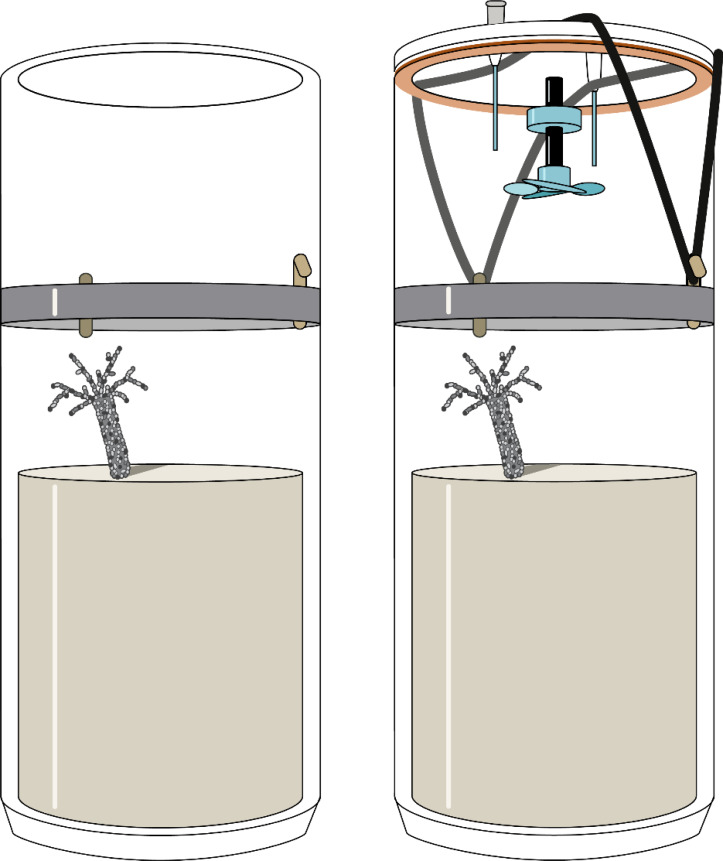
Experimental sediment cores used for the measurement of fluxes in the course of this study. The cores were open during the incubation (left) and only temporarily closed during flux measurements (right). The gas-tight lid was equipped with a magnetically driven stirrer and two capillaries for oxygen measurements and water sampling.

On day 8 and 9, gas-tight lids were added onto the cores (Fig. 1) and held in place by elastic bands. The lids were installed with a rubber gasket, and a small magnetic propeller on the underside. Two thin capillaries extended down into the core to enable the insertion of a fiber-optical oxygen optode, and to take water samples from the core (Fig. 1). The capillaries had Luer Lock connectors to draw water samples by syringes while the cores were submerged in the mesocosms. Along a time gradient ranging approximately 7 h, three samples to measure the oxygen concentration and nutrient turnover were taken per core. At each time point within each core, O_2_ and nutrient samples were collected. First, the water in each core was stirred with a magnetic attachment on a drill. The stirring was conducted at a threshold not strong enough to resuspend any sediment, but still strong enough to thoroughly mix the water column. The overlying water column in the incubations was not continuously stirred during flux sampling. Therefore, a small degree of stratification cannot be excluded. However, the overlying water column of 1L was comparably small, and previous studies investigating hypoxia effects on bioturbation (Beam et al., 2022), utilising similar aquaria, have demonstrated that the water column oxygen level only became stratified after 24 h. Therefore, potential stratification within our incubation cores would have been insignificant. Next, the concentration of oxygen (O_2_; µmol/L) was measured by inserting a fiber-optical oxygen optode though the capillaries (Firesting Fiber-Optical Oxygen Meter: PyroScience) into the core supernatant. The oxygen meter was calibrated using a two-point calibration, applied with air-saturated water (100%) and oxygen-free water (0%) that was prepared by addition of sodium sulfite (Na_2_SO_3_) (Neumann et al., 2021). To measure nutrients, samples were taken by a syringe (20 ml, B.Braun), filtered through a 0.45 µm cellulose filter (0.45 µm surfactant-free cellulose acetate membrane, Minisart Syringe Filter: Sartorius), stored in sterile containers (15 ml; Sarstedt) and refrigerated.

In between nutrient and oxygen measurements, the capillaries were closed with Luer Lock stoppers. Throughout the experiment, the tanks’ transparent lids allowed the natural light cycle to penetrate the incubation cores. Yet during the nutrient flux and oxygen incubation, the mesocosm tanks were covered in black sheeting to block out light, thus minimising photosynthetic activity. Due to time restrictions, there was no adjustment period of the incubation cores to the newly darkened mesocosm tanks prior to taking the nutrient samples. The O_2_ concentration was analysed during measurements, and measurements were stopped when the O_2_ concentration was less than 80% saturation. This ensured that the oxygen penetration depth in the sediment was not changed, which would have subsequently altered benthic fluxes, or drastically changed animals’ bioturbation behaviour. Further, as the measurement of fluxes itself could have introduced disruptions in the animals’ bioturbation behaviour caused by vibrations during the sampling and the darkening of the cores, the measurement was conducted only over the course of 1 day for each incubation core. Therefore, after the last time point was measured in each mesocosm tank, the core lids were promptly removed to allow water and oxygen exchange between the cores and overlying tank water once more.

Concentrations of different nutrients, total inorganic nitrogen (TIN), nitrate, (NO${}_{3}^{-}$), nitrite (NO${}_{2}^{-}$), ammonium, (NH${}_{4}^{+}$), silicate (SiO${}_{4}^{4-}$), and phosphate (PO${}_{4}^{3-}$), were sampled from the core water and measured with a continuous segmented flow autoanalyzer (SEAL Analytical HR3). The measured concentrations of nutrients in the overlying core water were corrected for the small amount of tank water introduced while sampling. A comparison of net fluxes revealed the introduction of water from the tank did not make a significant difference.

The net fluxes (J) of the nutrients and oxygen were then calculated using linear regression of concentrations over time (R), the volume of the supernatant of the cores (V), and the surface area of the cores (A) according to (1). Absolute fluxes from species cores can be viewed in Fig. S1 in the supplementary materials.

(1) \begin{eqnarray*}J= \frac{RV}{A} .\end{eqnarray*}

Oxygen and nutrient fluxes of the species cores were corrected by subtracting the average flux of the corresponding sediment control cores with respect to sampled site and temperature. This accounts for potential photosynthetic activity of any algae growing within the cores over the experimental time period. Further, by subtracting the control cores, macrofaunal fluxes are further isolated due to microbial and meiofaunal activity. Those fluxes attributed to microbial and meiofaunal activity will never be fully removed from the incubation cores, as there is often positive feedback between both macrofaunal activity, meiofaunal activity and microbial fluxes (Mermillod-Blondin et al., 2004). Through subtracting the control cores, we attempt to come close to the true macrofaunal-induced nutrient fluxes. The fluxes were then normalized by the AFDM of the specimens (unit: µmol/g^−1^ h^−1^ m^−2^). This correction for AFDM removes differences in fluxes that could arise from variation between biologically active macrofaunal biomass (*e.g.*, the shells of *C. edule* add biologically inactive mass). As natural densities of the chosen macrofauna vary across the Wadden Sea, this correction makes nutrient fluxes from our *in-situ* core densities more applicable across regions. Normalized TIN, PO${}_{4}^{3-}$ and SiO${}_{4}^{4-}$ nutrient fluxes were then additionally plotted against the oxygen flux, as here it is a direct measure of the animal activity; by plotting nutrient fluxes against the oxygen flux, we gained an overview of the elemental transport within the incubation cores. Suspended and recently sedimented particles represent major food sources for bioturbating macrobenthic organisms, and the initial elemental ratios of the ingested particles can subsequently determine the elemental ratios of excretions. Thus, data was combined from Amann (2013), Böckel (2015), Burson et al. (2016), Oehler et al. (2015a), and Oehler, Schlüter & Schückel (2015b) to establish the local elemental stoichiometry of particulate matter as a reference for the measured fluxes. It is further assumed that the oxidation of 1 mole of carbon consumes 1.5 moles of oxygen. Table S3 summarizes the hypothetical C:N:P:Si:O_2_ ratio of benthic fluxes if compounds were completely remineralized and recycled back in the water column. This ratio was then superimposed onto the fluxes to indicate graphically whether the bioturbating species caused strong deviations from the natural stoichiometry within the incubation cores.

### Bioturbation measurements

**Figure 2:**
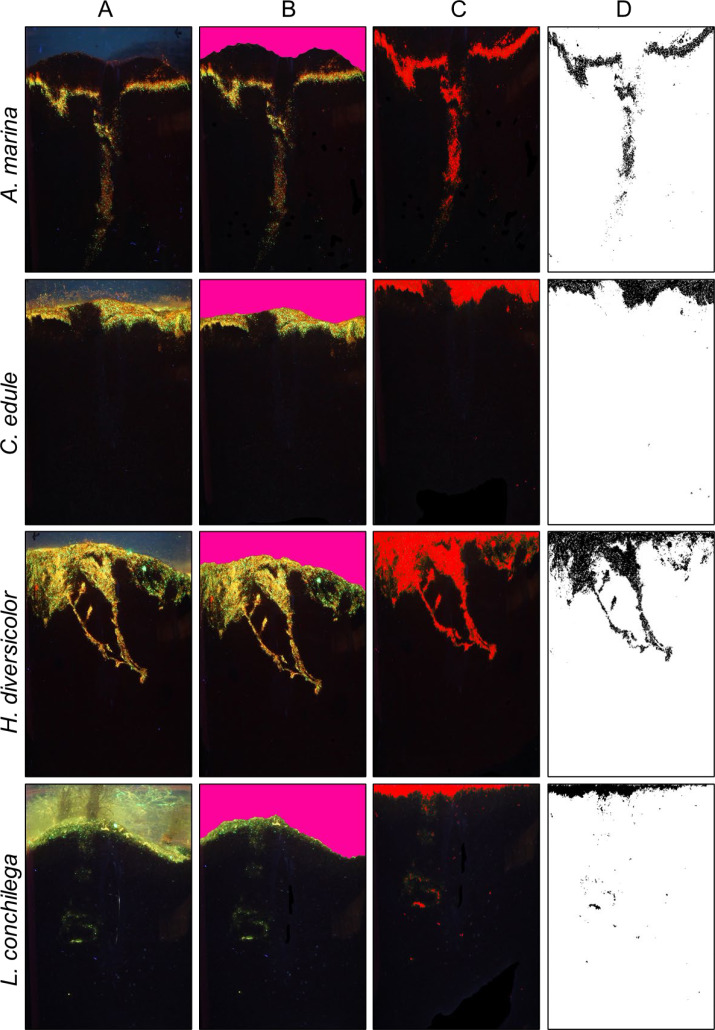
Bioturbation signatures of key species (*A. marina, *C. edule*, H. diversicolor, L. conchilega*). Image analysis process to calculate bioturbation rate of different species; (A) Initial photo of core (B) definition of sediment surface (C) selection of luminophores (D) isolation of luminophores for vertical profiling.

Bioturbation was measured by sediment profile imaging (SPI, Fig. 2) as previously described in Farrell et al. (2023) and Wrede et al. (2017). To take photos of the cores, cores were removed from the mesocosm tanks and placed in a black room. A blacklight (Phillips, TL-D 18W BLB 1SL) was used to illuminate the core sediment columns and luminophores. Photos were taken from a set distance of each side (180°) of the core (Camera: Canon EOS 500D, 15 mm, f 8, exposure 5.0 s, ISO 400). Afterwards, photos were also taken from the sides and from above under daylight.

Previous studies analysing bioturbation had utilised sediment slicing to recover luminophores (Maire et al., 2008), however Wrede, Holstein & Brey (2019b) experimentally demonstrated that differences in bioturbation measurements between these noted methods are insignificant for mobile fauna. Further, the possible distortion that may arise from the utilization of 2-dimensional images of rounded cylindrical objects is negligible (Wrede, Holstein & Brey, 2019b). Sediment profile imaging is preferable for looking at overall patterns of sediment reworking (Wrede, Holstein & Brey, 2019b), which is suitable here across the range of species used.

Images were analysed in ImageJ to ascertain bioturbation rates. All images taken were cut to the same size with the image analysis software Image J (1.52a) (https://imagej.nih.gov/ij/index.html). A specific uniform colour (Fig. 2B, RGB: 253, 003, 155; Gimp (2.10.20) (https://www.gimp.org/)) was used to manually colour the water column in images. ImageJ recognized this coloured water column through a custom-made plugin (available upon request), which furthermore removed the coloured water column and smoothed the sediment surface. A co-ordinate system was created, with the sediment-water interface as an ‘ *x* axis’, and the vertical sediment column the ‘ *y* axis’, using the custom-made plug-in. The highlighted luminophores within the images were then distinguished with the threshold function of ImageJ (Hue: 1–130, Saturation 1–250, Brightness: 60–255). The image was converted to black and white, with black pixels representing the luminophores (Fig. 2D). Each black pixel was counted within a pixel row, and transformed into a profile of luminophore distribution and depth. This image analysis method was used as formerly described in other studies, such as Farrell et al. (2023) and Wang et al. (2022). Through a non-linear regression analysis using the 1D diffusion model proposed by Crank (1975), the bioturbation rate (*D*_*b*_) was calculated (performed using Graph Pad Prism 5, GraphPad Software Inc.). This model can be applied for conservative tracers such as the luminophores, where no sedimentation occurs, and has been previously used in many bioturbation studies (for example; Farrell et al., 2023; Fernandes, Meysman & Sobral, 2006; Fernandes, Sobral & Costa, 2006; Maire et al., 2008; Wrede et al., 2017). Crank (1975) gave the solution as

(2) \begin{eqnarray*}C \left( x,t \right) = \frac{N}{\sqrt{\pi {D}_{b}t}} exp \left( \frac{-{x}^{2}}{4{D}_{b}t} \right) \end{eqnarray*}

where C(*x,t*) is the normalized tracer concentration relative to the initial input, *x* depth, N is the initial luminophore input, t is time, and D_b_ the biodiffusion coefficient, which is a measure for the bioturbation rate (Crank, 1975; Maire et al., 2008). This model assumes that luminophores are spread in an even layer at the sediment water interface.

The initial luminophore concentration N was estimated from the thickness (0.94) of the first layer of luminophore pixels from the luminophore profile data across all incubation cores.

Using this model, D_b_ was estimated by a non-linear regression fitted to the vertical profile of the luminophores, using the sum of least squares.

For our study we used the classical biodiffusion model from Crank (1975). This model emphasizes larger luminophore concentrations at the sediment surface (Fernandes, Meysman & Sobral, 2006), thus best fits the concentration data closer to the surface (<5 cm). Fortunately, the investigated species all predominantly carry out surficial sediment modification, so Crank’s model is used to cover the wide range of functional groups that the target organisms encompass. Any more direct, non-linear particle transport that occurs (*e.g.*, a luminophore particle dropping from the surface to the bottom of a burrow, or a *Lanice* worm transporting surface particles directly deeper into the sediment for tube building), is captured by the non-locality index (NLI).

The NLI calculates non-local transport, using the log-transformed luminophore tracer concentrations. This gives weight to lower concentrations; it uses the bioturbation rate of the actual tracer concentration (D_b_), and the calculated bioturbation rate from those same tracer concentrations after a log transformation (D${}_{\mathrm{b}}^{\mathrm{log}}$) (Fernandes, Meysman & Sobral, 2006). The measurement gives additional information regarding the variable sediment reworking that the bioturbators conduct, as the NLI gives more weight to lower particle concentrations by incorporating uninterrupted faster downward particle transport (Fernandes, Meysman & Sobral, 2006), as opposed to solely random short diffusive particle movement. The NLI is as follows:

(3) \begin{eqnarray*}NLI= \frac{ \left\vert {D}_{b}^{log}-{D}_{b} \right\vert }{\sqrt{{D}_{b}^{log}\times {D}_{b}}} .\end{eqnarray*}

If NLI = 0, the bioturbation rate of log-transformed tracer concentrations and the standard tracer concentrations are equal, demonstrating no non-local (non-diffusive) transport. Contrarily, a NLI >0 indicates varying levels of non-local transport of particles.

The mean weighted luminophore burial depth was calculated by multiplying the luminophore burial depth (cm) by the luminophore concentration at each depth.

The maximum luminophore burial depth was measured from the core images as the distance (cm) between the sediment surface and the deepest luminophore.

The bioturbation rates, maximum luminophore burial depths, and mean weighted luminophore burial depths of species cores were then corrected using the sediment control cores to eliminate the effect of two different sediment sites.

### Grain size

Sediment was dried in a drying oven (60 °C, 48 h), and then weighed. Samples were then sieved for 30 min in a Vibration-Sieve Machine (Fritsch Analysette, amplitude one mm), using a set of standard sieves (mesh sizes: two mm, one mm, 500 µm, 125 µm, 63 µm, and the pan). The weight of each fraction representing a particular grain size was then measured. The median grain size of the sediment from the Reef site was 408.2 µm, and 389.1 µm from the Uthörn site.

### Q_10_ calculation

The temperature co-efficient (Q_10_) for every 10 °C increase in temperature was calculated for the bioturbation rate and oxygen consumption of each species, using the following formula

(4) \begin{eqnarray*}{Q}_{10}={ \left( \frac{{R}_{2}}{{R}_{1}} \right) }^{ \frac{10}{({T}_{2}-{T}_{1})} }\end{eqnarray*}

Where R_1_ and R_2_ are either the bioturbation rates or O_2_ consumption measured at temperature 1 (T_1_:20 °C) and temperature 2 (T_2_:15 °C). The Q_10_ coefficients calculated for the species provide an estimate for the rate of change of a biological system, in this instance, sediment reworking of the bioturbating species as a result of an increase in temperature by 10 °C (Table 1), or the O_2_ consumption (Table 2) (Mundim et al., 2020). Generally, a Q_10_ value of 2 signifies a doubling in the measured process (Newell & Northcroft, 1967). Often the Q_10_ coefficient can alter in applicability to different biological processes, as they are not influenced by temperature alone (Salvato et al., 2001; Mundim et al., 2020). In this study however, it is a useful indication of how temperature dependent bioturbation and oxygen consumption processes differ between the cores of different bioturbating species. As such, we used the overall mean bioturbation and oxygen consumption rates across each group of species for each temperature, and calculated the Q_10_ from these averaged values (Tables S4 & S5).

**Table 1:** Q_10_ coefficients for bioturbation rates of species’ incubations.

	Q_10_
*Arenicola marina*	10.58
*Hediste diversicolor*	3.97
*Cerastoderma edule*	2.52
*Lanice conchilega*	0.84

**Table 2:** Q_10_ coefficients for oxygen consumption of species’ incubations.

	Q_10_
*Arenicola marina*	5.2
*Hediste diversicolor*	8.3
*Cerastoderma edule*	2.3
*Lanice conchilega*	2.5

### Flux extrapolation to species’ populations in the Sylt-Rømø Bight

As our experimental animals included juveniles, for an additional extrapolation of benthic fluxes evoked by the characteristic population of adult specimens of our chosen experimental species in the Wadden Sea, we calculated the average normalized fluxes based upon the average biomass of a population of each experimental species. These values were taken from the same location in the Sylt-Rømø Bight (Table S6; Baird, Asmus & Asmus, 2004), and also estimated the propagation of uncertainty. Baird, Asmus & Asmus (2004) calculate the biomass (g C m^−2^) of each experimental species populations’ using published literature from previous studies. AFDM values were calculated from Baird’s biomass estimates by using the conversion factor from Brey (2001) and can be viewed in Table S6. Here we made the assumption that the four species of this study are dominant within their Wadden Sea community on Sylt, and additively conjoined the 4 species to be a model ‘community’.

### Statistical analyses

For the variables describing sediment reworking, specifically D_b_, Lum_mean_, Lum_max_, and NLI, 2-way ANOVAs (*α* = 0.05, Table 3) were performed using ‘temperature’ and ‘species’ as fixed factors. The levels within the factor ‘temperature’ were 15 or 20 °C. The factor ‘species’ included 4 levels: *A. marina, C. edule, H. diversicolor,* or *L. conchilega*. Prior to the analysis, data was checked for normal distributions using a Shapiro–Wilk test. A Levene’s test was used to test for equal variances. The sediment reworking data did not meet the assumptions of normality or homogenous variances, therefore was subject to an aligned-rank transformation prior to the ANOVA using the R package ‘ARTool’ (version 0.11.1, Wobbrock et al., 2011), a robust non-parametric approach. For post-hoc analysis, Tukey’s multiple comparison test was used in analysis of levels within the single factors (species and temperature; Tables S7 & S8) (Kay & Wobbrock, 2020; Wobbrock et al., 2011). We explored the highest order significant interactions using the contrast test with the Holm *p*-value correction using the R package ‘phia’ (Table S9) (De Rosario-Martinez, 2015).

**Table 3:** Analysis of Variance of aligned-rank transformed data for main effects of bioturbation parameters. Significant values (*p* <0.05) are in bold.

Factors & interaction for each variable	df	df res.	F	*p*
**Bioturbation rate**				
Temperature	1	37	27.937	**<0.0001**
Species	3	37	42.125	**<0.0001**
Temperature × Species	3	37	10.253	**<0.0001**
**Non locality index**				
Temperature	1	37	2.95474	0.093984
Species	3	37	14.34220	**<0.0001**
Temperature × Species	3	37	0.83085	0.485450
**Mean weighted luminophore depth**				
Temperature	1	37	13.9402	**<0.0001**
Species	3	37	33.3231	**<0.0001**
Temperature × Species	3	37	8.2363	**<0.0001**
**Maximum luminophore burial depth**				
Temperature	1	37	1.0726	0.30707
Species	3	37	13.4304	**<0.0001**
Temperature × Species	3	37	1.4969	0.23136

To model the variables of the normalized O_2_, TIN, SiO${}_{4}^{4-}$, and PO${}_{4}^{3-}$ fluxes, generalized linear models (GLM) with an identity link function were used. This was due to the data not following a normal distribution, even after transformation attempts. Fixed factors were ‘temperature’ and the ‘species’, and the interaction between both. The model that best fit each dependent variable was chosen by backwards step-selection, and comparing Akaike’s Information Criterion (AIC) (Table S10, Field, Miles & Field, 2012). In order to assess whether the variables (temperature, species, and their interaction) were significantly predicting the different nutrient fluxes, a Wald-chi squared test was applied to the models that had been chosen by AIC (Table S11). The R package ‘car’ (version 3.1.1) was used to fit the models to the data (Fox & Weisberg, 2019).

To test significant differences of extrapolated fluxes for an adult population of the chosen species, *t*-tests were performed based on calculated averages (${\overline{X}}_{1}$) (Table S12), uncertainty (${s}_{1}^{2}$; including propagation of uncertainty), and sample size (*N*) by

(5) \begin{eqnarray*}t= \frac{{\overline{X}}_{1}-{\overline{X}}_{2}}{\sqrt{ \frac{{s}_{1}^{2}}{{N}_{1}} + \frac{{s}_{2}^{2}}{{N}_{2}} }} .\end{eqnarray*}

Lastly, to test whether there were significant differences in biomass between temperature treatments within species groups, AFDM data from incubation cores was checked for normality (Shapiro–Wilk test) and equal variances (Levene’s test), and Welch’s t-tests were calculated (Table S13). All statistical analysis was performed in R version 4.2.0, R Core Team (2022).

## Results

All organisms promptly began to bury within sediment cores when added to the mesocosm tanks. Throughout the experiment, the sediment colour slowly changed on the outside of cores from dark black-brown to a lighter browner colour, likely with oxidation of sediment around the outside of cores from photosynthetic activity. Characteristic bioturbation signatures of each species are illustrated in column A of Fig. 2. No significant differences between AFDM of animals were found within the different species groups (Table S13).

### Bioturbation parameters

**Figure 3:**
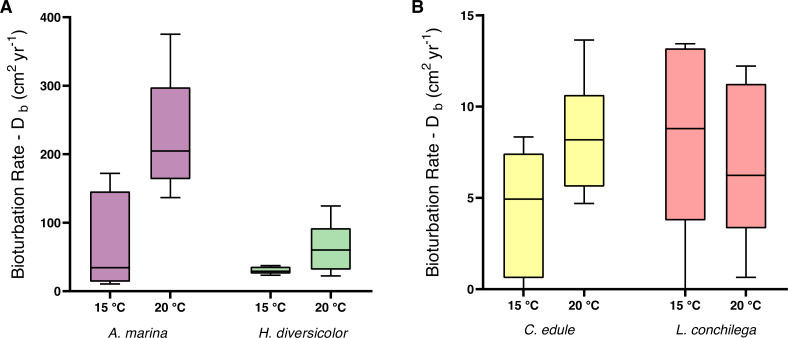
Bioturbation rate (cm^2^ yr^−1^) of (A) *A. marina* and *H. diversicolor* (B) *C. edule* and *L. conchilega*. Boxes represent lower and upper quartiles, lines indicate the median, and whiskers show minima and maxima.

Within the analysis of bioturbation rates, the bioturbation activity of the four tested species was differently affected by temperature (Table 3). *Arenicola marina* bioturbated significantly higher than all species, and this effect was amplified under 20 °C for the lugworm (each *p* <0.05; Fig. 3). The magnitude of the temperature effect also differed between the species. Thus, the difference between median bioturbation rates between the 15 and 20 °C temperature treatments for *A. marina* was 170.43 cm^2^ yr^−1^; demonstrating markedly more variation than 30.1 cm^2^ yr^−1^ for *H. diversicolor,* 3.24 cm^2^ yr^−1^ for *C. edule,* and only 2.56 cm^2^ yr^−1^ for *L. conchilega* (Fig. 3). This is reflected in the Q_10_ coefficients, where *L. conchilega* was the only species to have a Q_10_ coefficient <2 (Table 1).

Alongside the differential temperature effects, the investigated species also carried out varying levels of bioturbation (note the different scale of axes on Fig. 3). *Arenicola marina* conducted the most bioturbation, significantly more than the other species (each *p* <0.005, Fig. 3), whereas both *A. marina* and *H. diversicolor* conducted significantly more bioturbation than *C. edule* and *L. conchilega* (each *p* <0.0001, Fig. 3). As such, there was no significant difference between the bioturbation rates of *C. edule* and *L. conchilega* (*p* = 0.7757).

**Figure 4:**
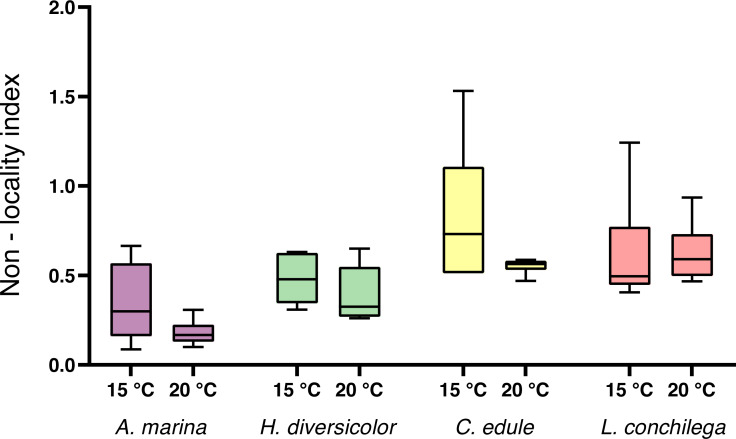
Non-locality index of *A. marina, H. diversicolor, C. edule*, and *L. conchilega*. Boxes represent lower and upper quartiles, lines indicate the median, and whiskers show minima and maxima.

For the non-local particle transport, estimated by the non-locality index (NLI), no significant interaction was detected between species and temperature (Table 3). There was a trend for a higher NLI in the 15 °C treatments (Fig. 4), however temperature was not found to be significant (Table 3). Nevertheless, species identity significantly affected the NLI (Table 3). *Cerastoderma edule* displayed the highest NLI, significantly higher than both *A. marina* and *H. diversicolor* (each *p* <0.05). On the other hand, *A. marina* exhibited the lowest NLI, significantly lower than *C. edule* and *L. conchilega* (each *p* <0.05). Similar to the bioturbation rate, there was no significant difference between the NLI of *C. edule* and *L. conchilega* (*p* >0.7136).

**Figure 5:**
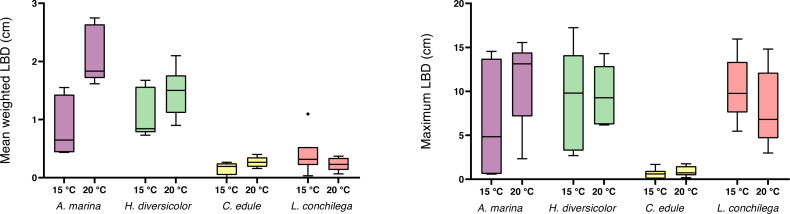
Mean weighted and maximum luminophore burial depth of cores containing *A. marina, H. diversicolor, C. edule*, and *L. conchilega*. Boxes represent lower and upper quartiles, lines indicate the median, and whiskers show minima and maxima.

A significant interaction was detected between temperature and species in the analysis of the mean luminophore burial depth, as the mean luminophore burial depth was differentially enhanced by the bioturbators under the two temperature regimes (Table 3). *Arenicola marina* buried luminophores deeper on average than *C. edule* and *L conchilega*, and this deeper burial was significantly furthered in the 20 °C treatment (each *p* <0.05; Fig. 5). Contrastingly, for *L. conchilega*, the mean luminophore burial depth was shallower in 20 °C treatment (Fig. 5). The mean luminophore burial depths between *A. marina* and *H. diversicolor,* and *C. edule* and *L. conchilega,* did not differ significantly between each other (*p* = 0.9705; *p* = 0.5612, respectively).

There was no significant interaction between temperature and species found for maximum luminophore burial depth, yet the factor species was significant (Table 3). *Cerastoderma edule* conducted shallower digging compared to all the other species (each *p* <0.05), with *C. edules*’ deepest luminophore burial measured at only 1.76 cm (Fig. 5). Although *L. conchilega* had some of the lowest bioturbation rates, there was no significant difference found between the maximum luminophore burial depths of *A. marina, H. diversicolor,* and *L. conchilega* (each *p*>0.05, Fig. 5). *Arenicola marina* buried the luminophores the deepest, with a trend for deeper burial at 20 °C. The deepest luminophore burial depth recorded across all species was achieved by *H. diversicolor,* at 17.24 cm (Fig. 5).

### Benthic fluxes

**Figure 6:**
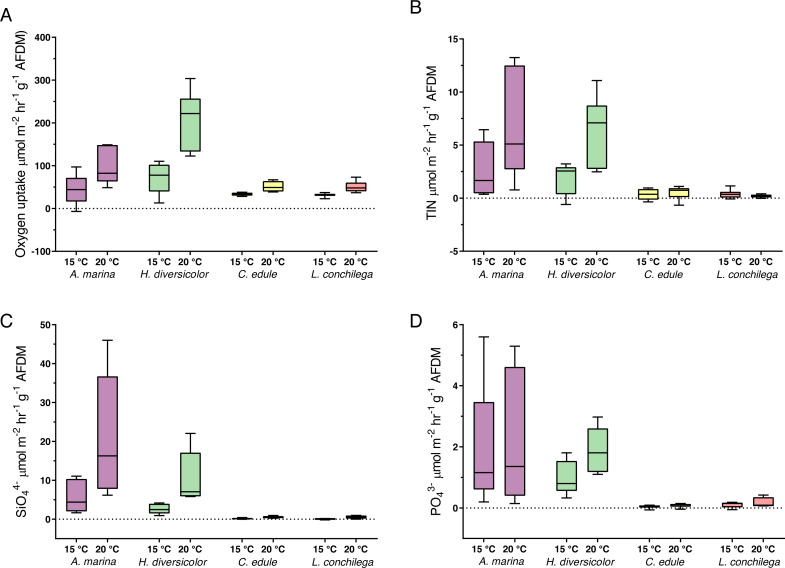
Nutrient fluxes ((A) Oxygen uptake; (B) TIN; (C) SiO${}_{4}^{4-}$; (D) PO${}_{4}^{3-}$; µmol m^−2^ hr^−1^ g^−1^ AFDM) from incubation cores (corrected by blank incubation cores and normalized by AFDM). Note that for the above nutrient plots (B, C, D) the dotted line highlights the border between the flux being positive (out the sediment) or negative (into the sediment). Note the difference of scales. Boxes represent lower and upper quartiles, lines indiciate the median, and whiskers show minima and maxima.

All bioturbators significantly increased sedimentary oxygen uptake, and this effect was furthered under 20 °C, as the oxygen uptake was significantly affected by the interaction between species and temperature treatment (Wald-Chi: *α* <0.05, Fig. 6A). Species thus increased oxygen uptake differently. *Arenicola marina* and *H. diversicolor* caused the strongest consumption of oxygen, with high O_2_ uptake; up to 149 µmol m^−2^ hr^−1^ g^−1^ AFDM and 303 µmol m^−2^ hr^−1^ g^−1^ AFDM, respectively, under the 20 °C treatment. The oxygen consumption in cores of these two species was contrastingly higher than those in cores with *C. edule* and *L. conchilega*. The latter two species caused similar oxygen uptake rates across temperature treatments (Fig. 6A).

The analysis of nutrient fluxes revealed varying species and temperature effects, but the effects did not always interact (Table S10). A significant interaction between the factors species and temperature was detected for the TIN and SiO${}_{4}^{4-}$ (Wald-Chi: *α* <0.05,) fluxes; therefore, *A. marina, C. edule, H. diversicolor* and *L. conchilega* all significantly enhanced the efflux of SiO${}_{4}^{4-}$ and TIN, and this effect was increased under 20 °C (Wald-Chi: *α* <0.05, Fig. 6). In fact, under 20 °C, *A. marina* nearly doubled the amount of SiO${}_{4}^{4-}$ efflux compared to any other species (Fig. 6C).

Only the species identity was significant in enhancing the PO${}_{4}^{3-}$ fluxes (Wald-Chi: *α* <0.05, Fig. 6D), with all species increasing the efflux of PO${}_{4}^{3-}$, regardless of temperature. There was, however, slightly more PO${}_{4}^{3-}$ efflux within *H. diversicolor* incubation cores under 20 °C (Fig. 6D).

**Figure 7:**
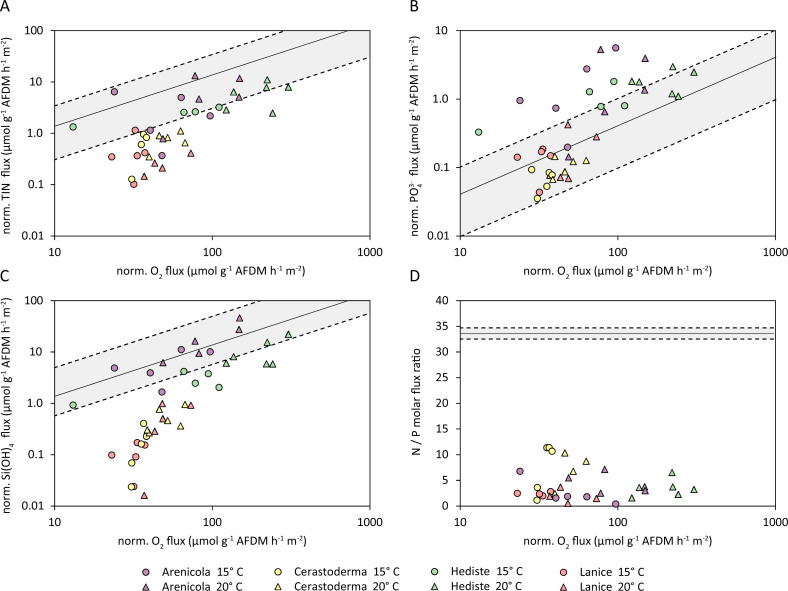
Normalized fluxes (µmol g^−1^ AFDM m^−2^ hr^−1^) of (A) Total inorganic nitrogen (TIN); (B) phosphate (PO${}_{4}^{3-}$); (C) silicate (Si(OH)_4_); and (D) N:P flux ratios, plotted against the normalized oxygen flux. The solid black line indicates the hypothetical regional elemental ratio as a reference, the dashed lines indicate the confidence band.

The TIN fluxes of all species virtually all fell below the hypothetical TIN:O_2_ ratio (Fig. 7A). The TIN fluxes from *A. marina* and *H. diversicolor* were generally within the confidence band of the TIN:O2 flux ratio, while the fluxes from *C. edule* and *L. conchilega* were significantly below this trend. Similarly, the normalized PO${}_{4}^{3-}$ fluxes approximately scaled with the normalized oxygen ratio; and although the measured PO${}_{4}^{3-}$ fluxes aligned well with oxygen flux for *C. edule* and *L. conchilega,* almost all PO${}_{4}^{3-}$ fluxes from *A. marina* and *H. diversicolor* substantially exceeded the hypothetical P:O_2_ trend (Fig. 7B). In contrast, silicate fluxes from *C. edule* and *L. conchilega* fell below the SiO${}_{4}^{4-}$:O_2_ trend, and silicate fluxes from *A. marina* and *H. diversicolor* agreed with the hypothetical ratio (Fig. 7C). The resulting ratios of N:P fluxes also deviated from the hypothetical regional ratio, ranging from up to 12 from *C. edule* to <1 in *L. conchilega* cores (Fig. 7D), yet all were below the hypothetical N:P ratio of 33.6 ± 1.1 for the area (Fig. 7D, Table S3).

**Figure 8:**
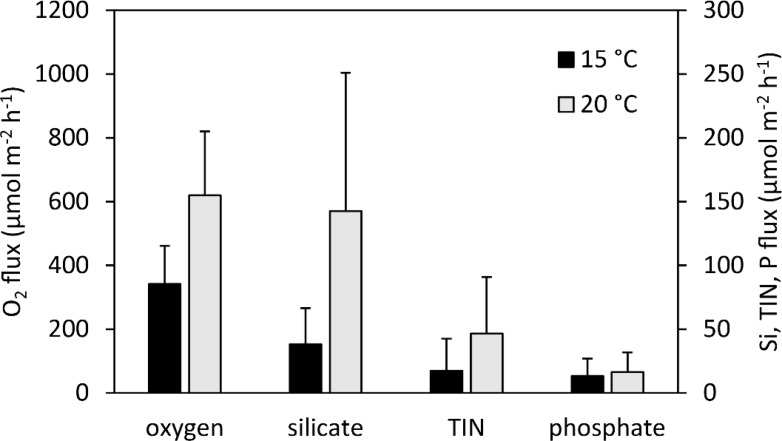
Estimated additively combined oxygen and nutrient fluxes of a characteristic Wadden Sea community made up of our experimental species (*A. marina, H. diversicolor, C. edule*, and *L. conchilega*). This is based upon normalized fluxes (this study) and representative benthic fauna data (AFDM) from Baird, Asmus & Asmus (2004) at 15 °C (black) and 20 °C (grey). Error bars represent 1 standard deviation including propagation of uncertainty.

In applying our normalized fluxes to the AFDM biomass values of the experimental species’ population in the Sylt-Romo Bight from Baird, Asmus & Asmus (2004), we found significantly increased fluxes in the 20 °C treatment (Fig. 8) for O_2_ (*p* = 0.004), SiO${}_{4}^{4-}$ (*p* = 0.023), and TIN (*p* = 0.054). Nutrient fluxes were increased approximately twofold for O_2_ and TIN, and even more than threefold for SiO${}_{4}^{4-}$ (Fig. 8).

## Discussion

### Benthic animals under warmer temperatures

The observed bioturbation activity rates in our study largely reflected species’ burrowing behaviours. The strongest sediment reworking was seen from *A. marina,* as its intense downward drawing of sediment during deposit feeding, and subsequent defecation on the sediment surface creates entire new sediment layers (Cadee, 1976). The strong bioturbation rates from *A. marina* were enhanced by temperature, also reflected in the Q_10_ value (10.58). This Q_10_ is biologically unrealistic, yet *A. marina* is known to enter anaerobic metabolism from 17–20 °C (Sommer, Klein & Pörtner, 1997), so the mesocosm temperature of 20 °C was likely close to a critical limit of 21 °C for *A. marina* (Sommer, Klein & Pörtner, 1997). Thus, the high Q_10_ value for its bioturbation rate (10.58) is likely a result of *A. marina* approaching its *pejus* temperature (Pörtner & Farrell, 2008; Sommer, Klein & Pörtner, 1997; Sommer & Pörtner, 1999), a point at which anaerobic products also begin to be suboptimally metabolized. The continual submersion in this experiment within 20 °C water may have brought the *A. marina* close to this threshold, so it is probable that they were bioturbating at their peak activity limits. Accordingly, sustained temperatures in the mesocosms beyond 20 °C could have impeded efficient bioturbation.

In line with high bioturbation rates, *A. marina* also displayed the strongest ability to facilitate nutrient exchanges between the sediment-water interface. The efflux of SiO${}_{4}^{4-}$ and TIN were especially pronounced, particularly under 20 °C. Silicate efflux is known to be exceptionally enhanced by temperature rise alone, as silicate solubility and dissolution rates are increased (Sigmon & Cahoon, 1997). The stark increase in silicate efflux by *A. marina* is in accordance with its observed bioturbation rates, as an increase in its irrigation current and deposition of fecal matter are concomitant with bioturbation (Retraubun, Dawson & Evans, 1996). Hence, as more water is ventilated throughout the sediment, and as larger surface areas of sediment are reworked and come into contact with the overlying water, effluxes are heightened. In addition, increased faecal deposition under 20 °C from *A. marina* would have contributed towards considerable ammonium efflux compared to other species (Henriksen, Rasmussen & Jensen, 1983). These large nutrient effluxes and bioturbation rates facilitate ecosystem functions and cement the role of *A. marina* as an ecosystem engineer, especially as the large release of ammonium is crucial for primary production (Laverock et al., 2011), and silicate important for benthic diatoms (Sigmon & Cahoon, 1997). Against this backdrop as a crucial ecosystem component, the fact that *A. marina* could have been working close to its physiological limits means that temperatures above 20 °C could hinder bioturbation and detrimentally affect primary productivity in the Wadden Sea.

Bioturbation by *H. diversicolor* was similarly stimulated by the temperature rise, conducting stronger bioturbation than previously noted under wider temperature conditions (17 °C–26 °C; 2–5 cm^−2^ yr^−1^) (Duport et al., 2006; Fernandes, Meysman & Sobral, 2006), albeit using slight variations of the same D_b_ coefficient. Further, sediment reworking and foraging temperature optimums have been found for *H. diversicolor* at 21 °C (Gillet et al., 2012) and 13–23 °C (Lambert et al., 1992), in line with our mesocosm tank temperatures. *Hediste diversicolor* is also known to increase sediment reworking with more chlorophyll content in the water column (Christensen, Vedel & Kristensen, 2000), so enhanced bioturbation rates for the polychaete likely came about from a comfortable temperature range in conjunction with high productivity within the Sylt-Rømø  Bight during the summer period. *Hediste diversicolor* constructs elaborate burrow networks, increasing the burrow density in upper sediments by lateral branching at five cm depth (Davey, 1994), and conducts strong irrigation of these burrows (Kristensen, 1983). This irrigation aids in the transport of nutrients out from the sediment, giving rise to stronger effluxes, as also seen for TIN and SiO${}_{4}^{4-}$ in *Hediste* cores. *Hediste diversicolor* also caused strongest uptake of oxygen, especially at 20 °C. Results here, as well as from previous studies, demonstrate that temperature enhances the sediment reworking rate of *H. diversicolor*. Yet this keystone species is detrimentally affected by the combination of temperature and acidification (Bhuiyan et al., 2021), thus despite the comfortable temperature range within this experiment the ragworm may have experienced some level of oxidative stress, potentially causing higher oxygen demands from the polychaete (Table 2). Findings regarding oxygen consumption in this study emphasize that even though warmer temperatures have been found as optima for *H. diversicolor*, this polychaete may be more susceptible than initially expected to abiotic changes in its environment.

Although the bivalve *C. edule* is considered a strong bioturbator (Verdelhos, Marques & Anastácio, 2015), it demonstrated smaller reworking rates compared to the two afore-mentioned polychaetes. This is probably due to its predominant behaviour—individuals of *C. edule* primarily shake their bodies from side to side to bury in the sand, but remain more or less still once buried, with their siphons exposed out of the sediment (Richardson, Ibarrola & Ingham, 1993). This was also observed in our experiment. Nevertheless, findings of bioturbation rates for *C. edule* here are considerably larger than previously experimentally measured at 14 °C (0.9–1.9 cm^2^ yr^1^; Mermillod-Blondin, François-Carcaillet & Rosenberg, 2005; Mermillod-Blondin et al., 2004), and the observed increase in bioturbation under 20 °C is in accordance with a previously reported temperature optimum for *C. edule* activity at 20–23 °C (Verdelhos, Marques & Anastácio, 2015), from a population in Portugal. This is also in line with known thermal limits for this species from the Wadden Sea, where strong mortality only occurs beyond 30 °C (Compton et al., 2007; Kristensen, 1957). Nutrient fluxes were generally lower compared to the tested polychaetes. Kauppi et al. (2018) attributed decreased PO${}_{4}^{3-}$ effluxes with shallow sediment reworking depth in *Cerastoderma glaucum* (Bruguiére, 1789). Through increasing oxygenation of surface sediments, iron is converted more readily to Fe^3+^, thus increasing the buffering capacity of surface sediments for the adsorption of PO${}_{4}^{3-}$to Fe^3+^. However, although this could have contributed to the observed smaller phosphate effluxes, the agreement of phosphate to oxygen flux ratios of *C. edule* with the regional, hypothetical stoichiometry suggests that phosphate fluxes may not have been substantially attenuated by adsorption to iron oxides.

Although many experiments on *L. conchilega* have been conducted with temperatures ranging from 10 to 18 °C (*e.g.*, Braeckman et al., 2010; Buhr, 1976; De Smet et al., 2016; Mestdagh et al., 2020; Ropert & Goulletquer, 2000) and temperature is known to regulate reproductive life history stages of *L. conchilega* (Keßler, 1963), a temperature optimum for *L. conchilega* sediment reworking activity is currently still unknown.

Nutrient fluxes observed in this study were not particularly high for *L. conchilega,* although oxygen uptake rates here and previous results suggest constant bioirrigation of *L. conchilega* (De Smet et al., 2016; Forster & Graf, 1995). Bioirrigators are generally thought to have stronger influence on nutrient cycling than bioturbators (Braeckman et al., 2010; Wrede et al., 2019a). Here, oxygen consumption by the macrofauna within our incubations could have limited the available oxygen for NH${}_{4}^{+}$ oxidation to NO${}_{3}^{-}$ and NO${}_{2}^{-}$ but the outcome may change when nutrient measurements would be carried out over a longer time period.

Past investigations have also shown high ammonium fluxes by *L. conchilega,* as well as differences in NH${}_{4}^{+}$ effluxes across seasons (Braeckman et al., 2010). Yet considering the low temperature dependency (Q_10_ 1.74) in oxygen consumption observed in *L. conchilega*, as well as the insignificant temperature effect upon sediment reworking, the fluxes seem in line with the patterns observed in this study for this tube building polychaete.

With respect to elemental transport in our incubation cores, *A. marina* and *H. diversicolor* appeared to foster the recycling of TIN in a ratio to oxygen that lies close to the hypothetical regional N:O_2_ ratio, while there was a significant TIN deficit observed from *C. edule* and *L. conchilega.* This could be due to stronger irrigation creating well-maintained oxygenated pockets in the sediment. These enable oxygen-dependent nitrification of excreted ammonium that is subsequently denitrified in adjacent anoxic sections of sediment. This coupled nitrification-denitrification has been demonstrated to account for extensive N loss (Marchant et al., 2016), and these oscillating redox conditions in the cores from strong irrigation could have enhanced these coupled processes. In contrast, *A. marina* and *H. diversicolor* may not provide conditions for this level of coupled nitrification-denitrification; the outflow of respired water from *A. marina* burrows is oxygen depleted, perhaps constraining nitrification. Nevertheless, the strong pumping behaviour of *A. marina* and *H. diversicolor* further flushed dissolved phosphate from porewater into the water column, at rates that exceed the hypothetical regional ratio. These two polychaetes are thus instrumental in intensifying the bentho-pelagic coupling between the sediment and water column. On the other hand, as phosphate fluxes evoked by *C. edule* and *L. conchilega* agree well with the regional P:O_2_ ratio, their excretions likely do not percolate through the sediment where phosphate could potentially be precipitated with iron oxides. This apparently lower exchange is supported by the low efflux of silicate, which is released in the sediment by dissolution of diatom shells. All in all, the resulting benthic fluxes have a very low N:P flux ratio, which is likely a combination of increased phosphate mobilization from *A. marina* and *H. diversicolor,* and a pronounced nitrogen loss by *C. edule* and *L. conchilega*.

The reworking of sediment by the benthic animals used in this study likely had a strong positive effect on microbial respiration. While we attempted to correct for this within our analyses, it is impossible to completely isolate the respiration of the macrofauna from microbial respiration. Biogenic structures caused by macrofauna can have strong effects on solute exchange in sediments (Mermillod-Blondin et al., 2004). This is especially the case when strong bioirrigation activity introduces electron acceptors like oxygen into the sediment (Chen et al., 2017), thereby enhancing bacterial diversity and numbers (Chen et al., 2017; Glud, 2008). This has particularly been documented with *H. diversicolor* (Mermillod-Blondin et al., 2004), and *A. marina* (Goñi Urriza et al., 1999). In addition, meiofaunal activity can also interfere with macrofaunal influence on bacterial communities (Lacoste et al., 2018). The current experiment was thus limited in its ability to completely unravel the different stages of community metabolism and fluxes across the varying trophic levels present in the incubation cores. Ultimately, the increased contribution of microbes to measured fluxes may still be attributed to the experimental macrofauna in some part, as the microbial contribution effect would be absent without the stimulating macrofaunal activity effect as the cause.

While the specimens of *L. conchilega* used in this experiment were within standard adult size range (Ziegelmeier, 1964), specimens of *A. marina, C. edule*, and *H. diversicolor* were notably smaller than standard adult size (Beukema, 1982; Beukema & De Vlas, 1979). Deductions from this data, albeit realistic and applicable to the Wadden Sea, likely underestimate the true contribution of these macrofaunal species to bioturbation and nutrient cycling. In adult field populations, bioturbation rates and benthic flux values are likely larger; an extrapolation on the biomass of 142 individuals by Valdemarsen et al. (2011) showed that a 10-fold increase in the biomass of *A. marina* could correspond to a 16-fold increase in reworking activity. This is also visible in fluxes within our extrapolation using adult population biomass data from Baird, Asmus & Asmus (2004), where fluxes increased at least 2-fold with the 5 °C temperature increase. This underlines the pivotal role these benthic species play within the Wadden Sea.

### Consequences of a warmer Wadden Sea

Our results demonstrate that an initial upregulation of bioturbation can lead to enhanced nutrient cycling, more remineralization as more oxygenated water is introduced into sediments, and enhanced primary productivity. The extrapolated benthic fluxes of adult populations of our experimental animals suggest that the benthic nutrient fluxes would increase approximately twofold by a warming from 15 °C to 20 °C (Fig. 8). This extrapolation has combined species effects. Yet due to species’ differential bioturbation impacts on sediments, as well as whether a system is dominated by either advective or diffusive transport (Mermillod-Blondin & Rosenberg, 2006), this simplification must be interpreted with caution. Notwithstanding, it is necessary to be able to scale up existing measured nutrient fluxes to an ecosystem (Fang et al., 2021). Therefore, ecosystem functioning in the Wadden Sea may not be directly detrimentally affected by sustained temperatures around 20 °C in summer. In fact, the bentho-pelagic coupling within the Wadden Sea ecosystem could even be strengthened. However, this acceleration is limited, and as temperature rises, more susceptible species could be lost as activity limits are reached (Pörtner & Farrell, 2008). Water temperatures already reach 20 °C and higher in the Wadden Sea, and a trend towards an increasing number of warmer days is evident (Amorim et al., 2023; Beukema & Dekker, 2020; Van Aken, 2008). The warming effects we summarize are not a scenario for a distant future as the transition is already taking place. Alterations in the sediment reworking activities of benthic species as they acclimate to these changes should be investigated.

*Arenicola marina’s* ability to adapt to temperatures higher than the critical temperature of 20 °C is poor (Sommer, Klein & Pörtner, 1997). As such, it could be the first species where its bioturbation could be detrimentally impacted by the predicted sustained warmer temperatures. This is especially pertinent as the lugworm holds overarching abundance in the Wadden Sea. *Arenicola marina’* s bioturbation maintains a favourable environment for itself by maintaining low sulphide concentrations (Volkenborn & Reise, 2006), and keeping sediment permeable and unclogged by organic matter (Volkenborn et al., 2007). Thereby the lugworms’ activity prevents mudflat expansion at a cost to sandflats (Volkenborn et al., 2007). Overall, the presence of *A. marina* holds important implications for ecosystem stability. For example, recently, a new invasive green alga, belonging to the *Vaucheria* genus (de Candolle 1801), has become established in the northern Wadden Sea (Reise, Michaelis & Rybalka, 2022b; Reise, Lackschewitz & Wegner, 2022a). Through feeding and burrowing, the lugworm is able to inhibit the establishment of young rhizoids, however once established, thick hummocks of *Vaucheria* effectively exclude *A. marina* (Reise, Lackschewitz & Wegner, 2022a). Consequently, bioturbation that keeps the sediments loose and sandy is largely excluded. A loss of dominant bioturbation activity could expedite the already noted expansion of these of these *Vaucheria* mats, potentially radically altering the Wadden Sea ecosystem. Nevertheless, this is still a relatively new phenomenon, and the influence of warming waters in the Sylt-Rømø Bight on the spread and establishment of this newly invasive algae are still largely unknown. As a result, the upshot effect of *A. marina’s* dominant bioturbation also comes into question and adds incentive to further monitor this dynamic ecosystem closely.

An extended temperature increase beyond what was used in this study would bring both *H. diversicolor* and *C. edule* into a comfortable bioturbation activity range, thereby ecosystem functions that are facilitated by the two species may even be favourably enhanced. While *C. edule* was the least active bioturbator and nutrient recycler in our experiment, fluxes observed from *H. diversicolor* corroborate its central role within the Wadden Sea ecosystem. Depending on abundances of *H. diversicolor*, the ragworm could compensate for lost bentho-pelagic links that may come about if bioturbation from *A. marina* is reduced.

Lastly, stable *Lanice* reefs are keystone structures in the Wadden Sea, offering settlement and refuge for a broad range of species’ larvae and small fish species (Qian, 1999; Van Hoey et al., 2008), as well as affecting current velocities in the benthic boundary layer (Eckman, Nowell & Jumars, 1981). *Lanice conchilega* did not demonstrate large temperature susceptibility in its bioturbation and nutrient cycling. In light of the predicted temperature rise, this could prove vital for the resilience of the Wadden Sea ecosystem. Yet, as more vulnerable species could be excluded, *L. conchilega* may only be able to partially compensate for lost bentho-pelagic links, as the facilitated nutrient fluxes were quite low in comparison to the efficient nutrient recyclers *A. marina* and *H. diversicolor*. By some measure, the capacity of *L. conchilega* to ventilate the sediment observed here was similar to the capacity of *A. marina* with respect to their oxygen consumption. Within the bounds of their reefs, the tube building and deposit feeding activity of *L. conchilega* could even counteract build-up of viscous organic matter in sands and encroaching mudflats. The protection of *Lanice* reefs as proposed by Braeckman et al. (2014) would be vital in this context. However, whether the strong pumping activity of *L. conchilega* can be sustained over longer periods of warmer sea temperatures remains to be elucidated.

## Conclusions

Temperature rises in the Wadden Sea could initially be beneficial for ecosystem services through an upregulation of bioturbation activity from key benthic fauna. The present study underpins the role of these bioturbating animals as ecosystem engineers within their environments, by linking how their bioturbation activity contributes to nutrient fluxes and subsequent bentho-pelagic coupling within the sediment. Additionally, both *A. marina* and *H. diversicolor* facilitate the recycling of major nutrients into the water column, which can subsequently fuel renewed primary production. Despite this amplified activity, bioturbation from both *A. marina* and *H. diversicolor* may demonstrate some sensitivity to sea temperatures rising for sustained periods of time, thereby limiting polychaete population numbers. This holds further implications for the Wadden Sea mudflats, both in terms of the food web and also geomorphologically, as changes in the makeup of the sandflats are observed. As the future of especially *A. marina*’s bioturbation activity comes into question, and amongst changes already observed in the Wadden Sea, the role of other dominant polychaetes like *H. diversicolor* and *L. conchilega* will likely become more important in maintaining vital faunal-mediated ecosystem functions.

##  Supplemental Information

10.7717/peerj.17047/supp-1 Supplemental Information 1 Supplemental figures and tables Biomass data and statistical information

10.7717/peerj.17047/supp-2 File S1 Raw luminophore burial depth measurements

10.7717/peerj.17047/supp-3 File S2 Raw oxygen and nutrient concentration measurements

## Additional Information and Declarations

### Data Availability

The following information was supplied regarding data availability: The raw data is available in the Supplemental Files.
