# Supplementary material for: Raised water temperature enhances benthopelagic links via intensified bioturbation and benthos-mediated nutrient cycling
Source: PeerJ. 2024 Feb 28;12:e17047. doi: 10.7717/peerj.17047 (PMC11636707; doi:10.7717/peerj.17047)
Supplement: Supplemental Information 1 — Biomass data and statistical information [file peerj-12-17047-s001.docx]

**Supplementary Materials**

**Raised water temperature enhances benthopelagic links via intensified bioturbation and benthos-mediated nutrient cycling**

**Authors: Eilish M. Farrell^1*^, Andreas Neumann^2^, Jan Beermann^1^, Alexa Wrede^1,2,3^**

**Table S1.** Temperature, salinity, and pH of the incoming water from the Sylt-Rømø Bight, prior to mesocosm temperature tank adjustment.

| Date | Mesocosm Tank | Temperature (degC) | pH | Salinity (psu) |
| --- | --- | --- | --- | --- |
| 24/08/2020 | A2 | 20.1 | 7 | 31.2 |
| 16:33 | B2 | 20.3 | 7 | 31.3 |
|  | C2 | 21 | 7 | 31.8 |
|  | D2 | 20.8 | 7 | 31.5 |
|  | E2 | 20.7 | 7 | 31.4 |
|  | F2 | 21.2 | 7.02 | 31.3 |
|  | A1 | 20.5 | 7 | 31.4 |
|  | B1 | 20.6 | 7.01 | 31.3 |
|  | C1 | 20 | 7 | 31.5 |
|  | D1 | 20.4 | 7 | 30.9 |
|  | E1 | 20.3 | 7 | 31.2 |
|  | F1 | 20.5 | 7 | 31.5 |
| 25/08/2020 | A2 | 20.3 | 7 | 31.2 |
| 09:33 | B2 | 20.1 | 7 | 31.3 |
|  | C2 | 20.4 | 7 | 31.7 |
|  | D2 | 20.2 | 7 | 31.7 |
|  | E2 | 19.6 | 7 | 31.5 |
|  | F2 | 20 | 7 | 31.4 |
|  | A1 | 20.3 | 7 | 31.3 |
|  | B1 | 20.8 | 7 | 31.4 |
|  | C1 | 20.6 | 7 | 31.6 |
|  | D1 | 20 | 7 | 31.5 |
|  | E1 | 20.3 | 7 | 31.4 |
|  | F1 | 20.1 | 7 | 31.6 |
| 26/08/2020 | A2 | 19.5 | 7 | 31.2 |
| 09:30 | B2 | 19.6 | 7 | 31.3 |
|  | C2 | 19.5 | 7 | 31.4 |
|  | D2 | 19.6 | 7 | 31.3 |
|  | E2 | 19.4 | 7 | 31.3 |
|  | F2 | 19.2 | 7 | 31.2 |
|  | A1 | 19.6 | 7 | 31.2 |
|  | B1 | 19.5 | 7 | 31.3 |
|  | C1 | 19.4 | 7 | 31.5 |
|  | D1 | 19.5 | 7 | 31.4 |
|  | E1 | 19.4 | 7 | 31.2 |
|  | F1 | 19.4 | 7 | 31.3 |
| 27/08/2020 | A2 | 19 | 7 | 31.1 |
| 09:30 | B2 | 19.1 | 7 | 31.3 |
|  | C2 | 19.2 | 7 | 31.4 |
|  | D2 | 18.7 | 7 | 31.4 |
|  | E2 | 18.5 | 7 | 31.2 |
|  | F2 | 18.6 | 7 | 31.1 |
|  | A1 | 19.4 | 7 | 31.1 |
|  | B1 | 19.3 | 7 | 31.2 |
|  | C1 | 19.3 | 7 | 31.4 |
|  | D1 | 18.6 | 7 | 31.3 |
|  | E1 | 18.9 | 7 | 31.2 |
|  | F1 | 18.8 | 7 | 31.5 |
| 28/08/2020 | A2 | 18.3 | 7 | 31.5 |
| 09:30 | B2 | 18.4 | 7 | 31.7 |
|  | C2 | 18.4 | 7 | 31.4 |
|  | D2 | 18.2 | 7 | 31.3 |
|  | E2 | 18 | 7 | 31.4 |
|  | F2 | 18.1 | 7 | 31.5 |
|  | A1 | 18.6 | 7 | 31.3 |
|  | B1 | 18.5 | 7 | 31.6 |
|  | C1 | 18.1 | 7 | 31.4 |
|  | D1 | 18.3 | 7 | 31.5 |
|  | E1 | 18.1 | 7 | 31.6 |
|  | F1 | 18.2 | 7 | 31.6 |
| 29/08/2020 | A2 | 18.6 | 7 | 31.3 |
| 09:30 | B2 | 18.7 | 7 | 31.2 |
|  | C2 | 18.9 | 7 | 31.4 |
|  | D2 | 18.5 | 7 | 31.7 |
|  | E2 | 18.4 | 7 | 31.7 |
|  | F2 | 18.3 | 7 | 31.8 |
|  | A1 | 18.5 | 7 | 31.5 |
|  | B1 | 18.6 | 7 | 31.4 |
|  | C1 | 18.7 | 7 | 31.5 |
|  | D1 | 18.3 | 7 | 31.3 |
|  | E1 | 18.2 | 7 | 31.6 |
|  | F1 | 18.5 | 7 | 31.4 |
| 30/08/2020 | A2 | 18.4 | 7 | 31.3 |
| 09:30 | B2 | 18.7 | 7 | 31 |
|  | C2 | 18.7 | 7 | 31.4 |
|  | D2 | 19.1 | 7 | 31.5 |
|  | E2 | 18.8 | 7 | 31.5 |
|  | F2 | 19 | 7 | 31.6 |
|  | A1 | 18.5 | 7 | 31.2 |
|  | B1 | 18.6 | 7 | 31.3 |
|  | C1 | 18.3 | 7 | 31.3 |
|  | D1 | 19.3 | 7 | 31.5 |
|  | E1 | 18.9 | 7 | 31.4 |
|  | F1 | 18.8 | 7 | 31.4 |
| 31/08/2020 | A2 | 18.6 | 7 | 31.1 |
| 09:30 | B2 | 18.8 | 7.02 | 31.3 |
|  | C2 | 19.3 | 7 | 31.4 |
|  | D2 | 19.1 | 7 | 31.3 |
|  | E2 | 19 | 7 | 31.5 |
|  | F2 | 19.2 | 7 | 31.4 |
|  | A1 | 18.7 | 7 | 31.3 |
|  | B1 | 19.5 | 7 | 31.6 |
|  | C1 | 19.3 | 7 | 31.7 |
|  | D1 | 18.2 | 7 | 31.8 |
|  | E1 | 19.1 | 7 | 31.7 |
|  | F1 | 18.5 | 7 | 31.3 |
| 01/09/2020 | not measured | | | |
| 02/09/2020 | A2 | 18.4 | 7 | 31.4 |
| 13:35 | B2 | 18.5 | 7 | 31.3 |
|  | C2 | 18.3 | 7 | 31.3 |
|  | D2 | 17.9 | 7 | 31.4 |
|  | E2 | 18.1 | 7 | 31.5 |
|  | F2 | 18 | 7 | 31.2 |
|  | A1 | 18.7 | 7 | 31.1 |
|  | B1 | 18.6 | 7 | 31.3 |
|  | C1 | 18.7 | 7 | 31.2 |
|  | D1 | 18.3 | 7 | 31.4 |
|  | E1 | 18.1 | 7 | 31.7 |
|  | F1 | 18.5 | 7 | 31.2 |

**Table S2.** Total biomass (Wet mass (g), dry mass (g), ash-free dry mass (g)), of incubation cores across species (A = *Arenicola marina*, H = *Hediste diversicolor*, C = *Cerastoderma edule*, L = *Lanice conchilega*). Cores with an asterisk were removed from analyses due to core contamination.

| Core | Temperature treatment (°C) | Mortality (%) | WM (g) | DM (g) | AFDM (Bio Active Mass) (g) |
| --- | --- | --- | --- | --- | --- |
| A1 | 15 | - | 0.16 | 0.112 | 0.068 |
| A2 | 15 | - | 1.13 | 0.791 | 0.185 |
| A3 | 15 | - | 1.17 | 0.799 | 0.24 |
| A4 | 15 | - | 0.42 | 0.25 | 0.136 |
| A5 | 15 | - | 0.52 | 0.441 | 0.093 |
| A6 | 15 | - | 0.53 | 0.298 | 0.18 |
| A7* | 20 | - | 0.4 | 0.215 | 0.162 |
| A8 | 20 | - | 0.42 | 0.255 | 0.121 |
| A9 | 20 | - | 1.33 | 0.651 | 0.275 |
| A10 | 20 | - | 0.37 | 0.261 | 0.085 |
| A11 | 20 | - | 1.12 | 0.528 | 0.254 |
| A12 | 20 | - | 0.99 | 0.491 | 0.24 |
| H1 | 15 | - | 0.65 | 0.221 | 0.098 |
| H2 | 15 | - | 0.7 | 0.22 | 0.079 |
| H3 | 15 | - | 0.76 | 0.185 | 0.071 |
| H4 | 15 | - | 0.86 | 0.19 | 0.081 |
| H5 | 15 | - | 0.53 | 0.164 | 0.071 |
| H6* | 15 | 2.08% | 0.19 | 0.042 | 0.025 |
| H7 | 20 | - | 0.59 | 0.134 | 0.064 |
| H8 | 20 | - | 0.72 | 0.151 | 0.059 |
| H9 | 20 | - | 0.66 | 0.18 | 0.076 |
| H10 | 20 | - | 0.9 | 0.246 | 0.08 |
| H11 | 20 | - | 0.73 | 0.187 | 0.09 |
| H12 | 20 | - | 0.76 | 0.253 | 0.08 |
| C1 | 15 | - | 7.97 | 5.302 | 0.48 |
| C2 | 15 | - | 9.32 | 6.081 | 0.594 |
| C3 | 15 | - | 7.57 | 5.263 | 0.426 |
| C4 | 15 | - | 8.33 | 5.576 | 0.538 |
| C5 | 15 | - | 8.32 | 5.394 | 0.488 |
| C6 | 15 | - | 7.86 | 5.414 | 0.486 |
| C7 | 20 | - | 9.24 | 6.317 | 0.596 |
| C8 | 20 | - | 9.64 | 6.647 | 0.526 |
| C9 | 20 | - | 7.8 | 5.453 | 0.415 |
| C10 | 20 | - | 7.76 | 5.362 | 0.474 |
| C11 | 20 | - | 8.33 | 5.628 | 0.508 |
| C12 | 20 | - | 8.41 | 5.918 | 0.544 |
| L1 | 15 | - | 2.16 | 0.844 | 0.631 |
| L2 | 15 | - | 2.89 | 1.207 | 0.668 |
| L3 | 15 | - | 3.53 | 1.239 | 0.771 |
| L4 | 15 | - | 3.11 | 1.233 | 0.716 |
| L5 | 15 | - | 2.48 | 0.974 | 0.543 |
| L6 | 15 | - | 2.2 | 0.833 | 0.48 |
| L7* | 20 | - | 3.86 | 1.465 | 0.7 |
| L8 | 20 | - | 2.89 | 0.922 | 0.616 |
| L9 | 20 | - | 2.97 | 1.056 | 0.601 |
| L10 | 20 | - | 2.61 | 0.873 | 0.66 |
| L11 | 20 | - | 2.84 | 0.782 | 0.502 |
| L12 | 20 | - | 1.94 | 0.543 | 0.468 |

**Table S3.** Molar ratio of benthic fluxes, based on elemental composition of suspended, particulate matter and surface sediment from the southern North Sea.

|  | **C** | **N** | **P** | **Si** | **O_2_** |
| --- | --- | --- | --- | --- | --- |
| average | 163.1 | 33.6 | 1.0 | 48.6 | 244.7 |
| 1 sd | 98.3 | 26.1 | 0.8 | 34.5 | 147.4 |

**Table S4.** Mean (± SD) bioturbation (D_b_) values for species incubation cores, used for the Q_10_ calculation.

|  | D_b_ 15 °C | Std. dev | D_b_ 20 °C | Std. dev |
| --- | --- | --- | --- | --- |
| *A. marina* | 70.26 | ± 69.38 | 228.57 | ± 89.50 |
| *H. diversicolor* | 33.56 | ± 5.58 | 66.89 | ± 36.34 |
| *C. edule* | 7.23 | ± 3.92 | 11.48 | ± 3.14 |
| *L. conchilega* | 10.75 | ± 5.51 | 9.85 | ± 4.47 |

**Table S5.** Mean (± SD) oxygen consumption values (O_2_, µmol/m^-2^ h^-1^ g^-1^ AFDM) for species incubation cores, used for the Q_10_ calculation.

|  | O_2_ 15 °C | Std. dev | O_2_ 20 °C | Std. dev |
| --- | --- | --- | --- | --- |
| *A. marina* | 44.35 | ± 35.20 | 101.13 | ± 45.06 |
| *H. diversicolor* | 72.37 | ± 37.13 | 207.93 | ± 67.89 |
| *C. edule* | 33.37 | ± 3.94 | 51.15 | ± 11.92 |
| *L. conchilega* | 31.80 | ± 4.74 | 50.03 | ± 13.74 |

**Table S6.** Biomass values used from Baird et al. (2004)

|  | Value from Baird et al. (2004) | Conversion factor from Brey (2001) | AFDM used for extrapolation |
| --- | --- | --- | --- |
|  | g C m^2^ |  | g AFDM m^2^ |
| *A. marina* | 5913.00 | 1.062 | 6.2796 |
| *C. edule* | 11400.00 | 1.447 | 16.4958 |
| *H. diversicolor* | 193.48 | 1.357 | 0.2626 |
| *L. conchilega* | 63.45 | 0.531 | 0.0671 |

**Table S7.** Tukey’s multicomparison post-hoc test of ART-transformed sediment reworking data for levels *within* the factor species (significance level *p* < 0.05, in bold).

| Contrast: | Estimate | Standard error | df | t.ratio | *p* |
| --- | --- | --- | --- | --- | --- |
| Bioturbation Rate |  | | | | |
| *A. marina: C edule* | 28.12 | 2.85 | 37 | 29.880 | **<.0001** |
| *A. marina: H.diversicolor* | 10.47 | 2.91 | 37 | 3.593 | **0.0050** |
| *A. marina: L. conchilega* | 25.40 | 2.91 | 37 | 8.720 | **<.0001** |
| *C. edule: H: diversicolor* | -17.65 | 2.85 | 37 | -6.202 | **<.0001** |
| *C. edule: L. conchilega* | -2.72 | 2.85 | 37 | -0.955 | 0.7757 |
| *H. diversicolor: L. conchilega* | 14.93 | 2.91 | 37 | 5.127 | **0.0001** |
| Non-locality Index |  | | | | |
| *A. marina: C edule* | -24.53 | 4.03 | 37 | -6.090 | **<.0001** |
| *A. marina: H.diversicolor* | -11.03 | 4.12 | 37 | 2.676 | 0.0516 |
| *A. marina: L. conchilega* | -20.25 | 4.12 | 37 | -4.911 | **0.0001** |
| *C. edule: H: diversicolor* | 13.50 | 4.03 | 37 | 3.351 | **0.0096** |
| *C. edule: L. conchilega* | 4.28 | 4.03 | 37 | 1.063 | 0.7136 |
| *H. diversicolor: L. conchilega* | -9.22 | 4.12 | 37 | -2.235 | 0.1326 |
| Mean luminophore burial depth |  | | | | |
| *A. marina: C edule* | 24.48 | 3.09 | 37 | 7.932 | **<.0001** |
| *A. marina: H.diversicolor* | 1.40 | 3.16 | 37 | 0.443 | 0.9705 |
| *A. marina: L. conchilega* | 20.43 | 3.16 | 37 | 6.468 | **<.0001** |
| *C. edule: H: diversicolor* | -23.08 | 3.09 | 37 | -7.479 | **<.0001** |
| *C. edule: L. conchilega* | -4.05 | 3.09 | 37 | -1.312 | 0.5612 |
| *H. diversicolor: L. conchilega* | 19.03 | 3.16 | 37 | 6.025 | **<.0001** |
| Maximum luminophore burial depth |  | | | | |
| *A. marina: C edule* | 19.78 | 4.14 | 37 | 4.777 | **0.0002** |
| *A. marina: H.diversicolor* | -2.07 | 4.24 | 37 | -0.488 | 0.9614 |
| *A. marina: L. conchilega* | -1.97 | 4.24 | 37 | -0.464 | 0.9664 |
| *C. edule: H: diversicolor* | -21.85 | 4.14 | 37 | -5.276 | **<.0001** |
| *C. edule: L. conchilega* | -21.75 | 4.14 | 37 | -5.252 | **<.0001** |
| *H. diversicolor: L. conchilega* | 0.10 | 4.24 | 37 | 0.024 | 1.0000 |

**Table S8.** Tukey’s multicomparison post-hoc test of ART-transformed sediment reworking data for levels *within* the factor temperature (significance level *p* < 0.05, in bold).

| Contrast: | Estimate | Standard error | df | t.ratio | P value |
| --- | --- | --- | --- | --- | --- |
| 15 °C – 20 °C |  | | | | |
| Bioturbation rate | -16.7 | 3.15 | 37 | -5.286 | **<0.0001** |
| Non-locality index | 6.93 | 4.03 | 37 | 1.719 | 0.0940 |
| Mean weighted luminophore burial depth | -13.6 | 3.65 | 37 | -3.734 | **0.0006** |
| Maximum weighted luminophore burial depth | -4.34 | 4.19 | 37 | -1.036 | 0.3071 |

**Table S9.** Contrast interaction test utilising the Holm *p*-value adjustment method (only completed where a significant interaction was found; significance level *p* < 0.05, in bold).

| Contrast: | value | Standard error | df | Sum of square | F | *p* |
| --- | --- | --- | --- | --- | --- | --- |
| Bioturbation rate |  | | | | | |
| 15-20°C:*A. marina-C. edule* | -39.77 | 8.581 | 1.0 | 2259.13 | 21.474 | **0.0002** |
| 15-20°C*:A. marina-H. diversicolor* | -25.133 | 8.783 | 1.0 | 861.39 | 8.1879 | **0.0276** |
| 15-20°C*:A. marina-L. conchilega* | -43.600 | 8.783 | 1.0 | 2592.22 | 24.6402 | **<0.0001** |
| 15-20°C*:C. edule-H. diversicolor* | 14.633 | 8.581 | 1.0 | 305.91 | 2.9078 | 0.1931 |
| 15-20°C:*C. edule-L. conchilega* | -3.833 | 8.581 | 1.0 | 20.99 | 0.1995 | 0.6570 |
| 15-20°C:*H. diversicolor-L. conchilega* | -18.467 | 8.783 | 1.0 | 465.02 | 4.4203 | 0.1271 |
| Residuals |  | 37.000 | 3892.5 |  |  |  |
| Mean luminophore burial depth |  | | | | | |
| 15-20°C :*A. marina-C. edule* | -36.300 | 9.162 | 1.0 | 1882.41 | 15.6966 | **0.0016** |
| 15-20°C *:A. marina-H. diversicolor* | -23.333 | 9.378 | 1.0 | 742.42 | 6.1907 | 0.0670 |
| 15-20°C :*A. marina-L. conchilega* | -42.933 | 9.378 | 1.0 | 2513.55 | 20.9593 | **0.0003** |
| 15-20°C:*C. edule-H. diversicolor* | 12.967 | 9.162 | 1.0 | 240.19 | 2.0028 | 0.3307 |
| 15-20°C :*C. edule-L. conchilega* | -6.633 | 9.162 | 1.0 | 62.86 | 0.5241 | 0.4736 |
| 15-20°C *:H. diversicolor-L. conchilega* | -19.600 | 9.378 | 1.0 | 523.85 | 4.3682 | 0.1306 |
| Residuals |  | 37.000 | 4437.2 |  |  |  |

**Table S10.** Best (lowest AIC) generalized linear models of Total Inorganic Nitrogen (TIN), Phosphate (PO_4_^3-^), Silicate (SiO_2_), and (O_2_). Colons (:) indicate interactions, whereas plus signs (+) indicate additive effects.

| Nutrient | Best model | AIC value |
| --- | --- | --- |
| Total Inorganic Nitrogen | TIN ~ Temperature + Species + Temp:Species | 214.94 |
| Phosphate | PO_4_ ~ Species | 139.05 |
| Silicate | SiO_2_ ~ Temperature + Species + Temp:Species | 299.02 |
| Oxygen | O_2_ ~ Temperature + Species + Temp:Species | 456.08 |

**Table S11.** Wald chi-squared test of the best (lowest AIC) generalized linear models of total inorganic nitrogen (TIN), phosphate (PO_4_^3-^), and silicate (SiO_2_) and oxygen (O_2_) as a function of the Species (Spp.), the Temperature treatment (Temp.) and the interaction (Int.) between the two. Dashes indicate that the factor was not included in the best model. The significance level was set to α < 0.05, and significant values are in bold.

|  | χ^2^ | | | *df* | | | *p* < χ ^2^ | | |
| --- | --- | --- | --- | --- | --- | --- | --- | --- | --- |
|  | Temp. | Spp. | Int. | Temp. | Spp. | Int. | Temp. | Spp. | Int. |
| TIN | 9.776 | 33.157 | 10.232 | 1 | 3 | 3 | **0.002** | **<0.001** | **0.017** |
| PO_4_^3-^ | - | 30.081 | - | - | 3 | - | - | **<0.001** | - |
| SiO_2_ | 10.624 | 32.675 | 11.733 | 1 | 3 | 3 | **<0.001** | **<0.001** | **0.008** |
| O_2_ | 29.268 | 63.314 | 21.221 | 1 | 3 | 3 | **<0.001** | **<0.001** | **<0.001** |

**Table S12.** t.test for differences in extrapolated nutrient fluxes between temperature using our flux data and biomass from Baird et al. (2004).

|  | test statistic | *df* | *p* |
| --- | --- | --- | --- |
| Oxygen | 4.204 | 5 | 0.004 |
| Silicate | 2.450 | 5 | 0.023 |
| TIN | 1.949 | 5 | 0.054 |
| Phosphate | 1.059 | 5 | 0.169 |

**Table S13.** t.test for differences in the ash-free dry mass (g) between temperature treatments (within each species group; significance level *p* < 0.05).

|  | test statistic | *df* | *p* |
| --- | --- | --- | --- |
| *A. marina* | -0.96244 | 7.2982 | 0.3666 |
| *C. edule* | -0.24674 | 9.9445 | 0.8101 |
| *H. diversicolor* | 0.76045 | 8.7491 | 0.4670 |
| *L. conchilega* | 1.1451 | 8.9325 | 0.2819 |

**Figure S1.** Absolute fluxes of (A) oxygen consumption (O_2_); (B) total inorganic nitrogen (TIN); (C); silicate (SiO_4_^4-^) (D); phosphate (PO_4_^3-^) (µmol m^-2^ hr^-1^) within species’ incubation cores. Note that the dotted line highlights the border between the flux being positive (out of the sediment) or negative (into the sediment). Note the difference of scales.

**Figure S2.** Absolute fluxes of (A) oxygen (O_2_); (B) total inorganic nitrogen (TIN); (C); silicate (SiO_4_^4-^) (D); phosphate (PO_4_^3-^) (µmol m^-2^ hr^-1^) within sediment control incubation cores. Note that the dotted line highlights the border between the flux being positive (out of the sediment) or negative (into the sediment). Note the difference of scales.
